# Therapeutic itineraries and explanations for tuberculosis: an indigenous perspective

**DOI:** 10.1590/S0034-8910.2015049005904

**Published:** 2016-01-14

**Authors:** Laura Maria Vidal Nogueira, Elizabeth Teixeira, Paulo Cesar Basta, Maria Catarina Salvador da Motta

**Affiliations:** I Departamento de Enfermagem Comunitária. Universidade do Estado do Pará. Belém, PA, Brasil; II Departamento de Endemias Samuel Pessoa. Escola Nacional de Saúde Pública Sérgio Arouca. Fundação Oswaldo Cruz. Rio de Janeiro, RJ, Brasil; III Departamento de Saúde Pública. Escola de Enfermagem Anna Nery. Universidade Federal do Rio de Janeiro. Rio de Janeiro, RJ, Brasil

**Keywords:** Tuberculosis, Indigenous Population, Medicine, traditional, Health Services, indigenous, Health Knowledge, attitudes, practice, Qualitative Research

## Abstract

**OBJECTIVE:**

To analyze explanations for tuberculosis and therapeutic itineraries of Brazilian indigenous people.

**METHODS:**

Case study with a qualitative-descriptive approach. We conducted semi-structured interviews with 11 Munduruku indigenous, including direct observation of treatment for tuberculosis in the municipality of Jacareacanga, south-western region of the state of Para, Brazil. To identify explanations for tuberculosis and therapeutic itineraries, we performed thematic content analysis.

**RESULTS:**

Traditional medicine was the first therapeutic option chosen by the indigenous. However, biomedicine was also employed, which indicates a circulation between different therapeutic contexts and health concepts among the Munduruku. The explanations provided ranged from recognition of the signs and symptoms specific to tuberculosis to the attribution of the disease to a spirit that leaves the body and wanders in the woods, returning ill into the body. Unlike the biomedical model, which links tuberculosis transmission strictly to interpersonal contact, in closed spaces without natural lighting and ventilation (preferably domestic environments), the Munduruku associate the disease to an indirect contact between people socially distant (enemies or adversaries) in public and open places.

**CONCLUSIONS:**

The explanations made by the indigenous are unique and deserve the attention of those who are responsible for developing health public policies, as well as of the teams who work on the villages. To guarantee an efficient control of tuberculosis in these regions, it is necessary that the developed actions integrate biomedicine knowledge and the traditional medicine of the indigenous people, in addition to respecting and welcoming local culture manifestations.

## RESUMO

**OBJETIVO:**

Analisar explicações sobre adoecimento por tuberculose e itinerários terapêuticos de indígenas brasileiros.

**MÉTODOS:**

Estudo de caso, com abordagem quali-descritiva. Foram realizadas entrevistas semiestruturadas com 11 indígenas Munduruku, incluindo observação direta do tratamento da tuberculose no município de Jacareacanga, região sudoeste do estado do Pará, em 2010. A fim de identificar explicações para tuberculose e itinerários terapêuticos, realizou-se análise de conteúdo temático.

**RESULTADOS:**

A medicina tradicional foi o itinerário terapêutico inicial escolhido pelos indígenas. Entretanto, a biomedicina também foi utilizada, indicando haver circulação entre diferentes contextos terapêuticos e concepções de saúde entre os indígenas Munduruku. As explicações variaram do reconhecimento de sinais e sintomas específicos da tuberculose até o relato de que o espírito sai do corpo, vagando pela floresta e que, quando retorna para o corpo, traz consigo a doença. Ao contrário do modelo biomédico, que vincula a transmissão da tuberculose exclusivamente ao contato interpessoal, em espaços fechados, sem luz natural e sem ventilação (preferencialmente em ambientes domésticos), a perspectiva Munduruku associa a doença ao contato indireto entre pessoas socialmente distantes (inimigos ou adversários) em locais públicos e abertos.

**CONCLUSÕES:**

As explicações formuladas pelos indígenas são singulares e merecem a atenção dos formuladores de políticas de saúde, assim como das equipes que atuam nas aldeias. Para garantir controle eficaz da tuberculose nessas localidades, é necessário que as ações desenvolvidas integrem conhecimentos da biomedicina e da medicina tradicional indígena, além de respeitar e acolher as manifestações da cultura local.

## INTRODUCTION

Interest in the impact of tuberculosis (TB) on Brazilian indigenous populations has increased within the past decades. Those populations have a high degree of exposure to *Mycobacterium tuberculosis* and a tenfold higher incidence rate compared with the national average.^7^ ^,^ ^9^ ^,^ ^18^ Moreover, the occurrence of drug resistance has been reported,^3^ as the significant prevalence of latent TB infection (LTBI),^6^ ^,^ ^4^ ^,^ ^21^ and many cases among children and adolescents.^5^ ^,^ ^17^

According to data provided by the Brazilian Ministry of Health, the overall incidence of TB in Brazil was 36.0/100,000 inhabitants in 2011, with higher rates in the Northern region (45.2/100,000), particularly in the state of Para (47.3/100,000).^a^ The incidence of TB reached 132.5/100,000 among the indigenous populations residing in this state in 2009.^b^

This indicates that TB represents a serious health problem for the Brazilian indigenous populations. Despite an increased interest in this theme, little is known about the dynamics of TB in indigenous villages, the explanations for the occurrence of the disease formulated by indigenous people, and the therapeutic itineraries chosen by TB patients.^9^ ^,^ ^10^ ^,^ ^15^

A recent literature review^8^ did not find studies that assessed the therapeutic itineraries of TB patients in indigenous populations.^8^ However, knowledge of the itineraries of people seeking health care may contribute to understand the behavior related to health care and use of health services.^1^ ^,^ ^8^ ^,^ ^16^ Also, it might contribute to the effectiveness of the strategies formulated for disease control. Such knowledge might serve as the basis for actions culturally adapted to the different realities of the various Brazilian indigenous groups.

This study aimed to analyze explanations for the occurrence of TB and the therapeutic itineraries of Brazilian indigenous people.

## METHODS

The Brazilian Unified Health System (SUS) includes a *Subsistema de Atenção à Saúde Indígena* (SASI – Indigenous Health Care Subsystem), established by the Law 9,836/1999. This subsystem comprises 34 *Distritos Sanitários Especiais Indígenas* (DSEI – Special Indigenous Sanitary Districts), which are linked to the *Secretaria Especial da Saúde Indígena* (SESAI – Special Secretary of Indigenous Health Care), under the Ministry of Health. Within the scope of DSEI, health care is provided at health care basic units, centers, and at *Casas de Apoio à Saúde do Índio* (CASAI – Indigenous Health Support Shelters).

The state of Para has four DSEI, from which Rio Tapajós DSEI was selected for the present study. The Rio Tapajós DSEI is the most populous of Para state. Although it covers 118 villages, most health actions are concentrated in the Jacareacanga municipality (06º13’20”S; 57º45’10”W), where most indigenous populations receive care from this DSEI.

Jacareacanga has 14,103 inhabitants, of whom 41.4% define themselves as indigenous.^c^ According to the *Sistema de Informações da Atenção à Saúde Indígena* (SIASI – Indigenous Health Care Information System), the Rio Tapajós DSEI provides care to 9,516 individuals from the Apiakám, Kaiapó, and Munduruku ethnic groups.^d^

The Munduruku are Tupi-speaking people, most of whom live in the municipalities of Itaituba, Novo Progresso, and Jacareacanga, in Para, Northern Brazil. Smaller groups live in Amazonas, Northern Brazil, and Mato Grosso, Midwestern Brazil, states.

The Munduruku numbered 11,630 persons (approximately 85.0% of the population assisted by the Rio Tapajós DSEI),^d^ living in a total of 14 Indigenous Territories at the margins of Tapajos and Amazonas rivers and near to Transamazônica road. The lands are covered by interfluvial tropical rainforest.

After the emancipation of the municipality by the Law 5,691/1991, indigenous people began to play an important social role in the local community, including positions in the executive and legislative spheres. However, land conflicts with farmers and miners are still frequent. Recently, the Munduruku have faced problems with large government projects, especially the construction of hydroelectric dams and waterways.

The Munduruku are organized by work cooperatives, mainly employed in flour production activities, chestnut collection and, in few communities, rubber production. The indigenous presence in the municipality is marked not only by the Munduruku living in urban areas, but also by villagers that frequently go there to seek health care, receive social benefits, and acquire industrial goods (such as salt, sugar, soap, clothes, fuel, and others).

Basic health care for indigenous people is locally available in the villages. Health care in other levels of complexity should be offered by the municipalities. Control of TB is mainly implemented at the primary care network level and includes: medical and nursing consultations; requests for complementary diagnostic tests; treatment indications; distribution of medicines; treatment monitoring; and final discharge.

This investigation used a qualitative-descriptive approach, based on semi-structured interviews. The themes selected to guide the interviews were based on everyday health-care practices, using the therapeutic system proposed by Kleinman^13^ as reference. The Kleinman system characterizes different types of health-care practices, which might be distributed across three sectors: the professional sector (formal medical practice – biomedicine); the popular sector (family-based and self-care practices); and the folk sector (mystical and religious practices).

The present study was conducted in the following villages: Katõ (n = 461), Missão São Francisco (n = 675), and Sai Cinza (n = 867). The villages were selected (probabilistic methods were not used) because of their population size (the three together comprise approximately 25.0% of the Munduruku Indigenous in Para and 35.0% of the population self-declared as indigenous in the Jacareacanga municipality) and because they concentrate the largest number of TB cases notified in the past four years, according to data provided by the Rio Tapajós DSEI.^e^

The informants were 11 indigenous patients, three of whom were under treatment, and eight had been treated for TB within the past four years. We selected both individuals undergoing and having completed treatment to broaden the scope of the narratives from June to August of 2010. Because the treatment consists of a daily intake of medication over 180 days, we assumed that it leaves marks or memories that might be recovered in directed interviews. The four-year limit was established to minimize memory bias. The interviewees’ ages varied from 20 to 84 years; six were male, and five female.

Three types of records were used to select the participants: record book of the Tuberculosis Control Program of Jacareacanga Municipal Hospital; health files at CASAI; and clinical records from the villages’ health care units.

The interviews were conducted in two settings: the informants under treatment were interviewed at CASAI, and the subjects who had completed treatment were interviewed in the villages. The principal investigator conducted all interviews. The interview script was previously tested with a sample from a different ethnic group.

Nursing technicians and indigenous health agents participated in the interviews as interpreters in seven cases. The translations were simultaneous, occurring immediately after each question and answer. The narratives were recorded using a digital voice recorder and then fully transcribed. The participants were identified using three consonants indicating their ethnicity (MDK) followed by a number that represents their place in the sequence of interviews.

Data systematization and analysis were performed according to the thematic content analysis technique formulated by Bardin,^2^ which allowed for analyzing the narratives based on the identification of the meanings included in each coding unit. Record units were identified and their absolute frequency was estimated, the context units were identified, and categories were constructed, which facilitated reconstructing the narratives’ meaning.^2^

This study is part of a larger project titled “Magnitude of Tuberculosis and Therapeutic Itineraries of the Munduruku in Para, Brazilian Amazonia”. This study was approved by the research ethics committee of Universidade do Estado do Pará and the National Commission of Research Ethics (Process 116/2010), and was authorized by the National Indigenous Foundation (Process 0724/2010).

## RESULTS

The analysis of narratives led to the elaboration of two categories that summarize the Munduruku explanations for illness. One category concerned the indigenous patient knowledge of the disease, the symptoms they were able to recognize in their own bodies, and their ideas regarding the transmission of TB. The other category concerned their personal experience with TB and thus described the therapeutic itineraries established to solve the problem posed by the disease.

The first category concerned concrete and imaginary features of TB. The explanations provided by the Munduruku for TB were based on a dual perspective that ranged from the concrete to the abstractive one. The concrete perspective was mainly identifiable in narratives in which associations were established between the disease and the signs and symptoms that the participants were able to recognize in their own bodies. Chest pain and cough were predominant (mentioned in nine and eight narratives, respectively). According to the participants, “Tuberculosis is a disease like… night cough, fever, and chest pain” (MDK-11)*.* Self-perception of being ill was manifested as pain and physical suffering: “Tuberculosis is a disease that causes chest pain, backache, mucus, weakness” (MDK-5). “[…] It is a very dangerous disease; it gives chest pain, headache, and fever” (MDK-1).

Only unspecific bodily manifestations were reported in some narratives: “It all began with pain in the legs; it went upwards, and then cough appeared… I couldn’t sleep at night […]” (MDK-7), which denotes a personal interpretation regarding the signs, symptoms, and transmissibility of TB.

Some interviewees reported an association between the disease and work-related activities (corresponding to the daily routine in the village) that demand strong physical exertion: “I felt tuberculosis because I worked really hard carrying logs and firewood” (MDK-1). “I got it because I roasted flour in a hot oven […]” (MDK-9).

Concerning the abstract perspective, the Munduruku admitted that TB is “[…] a very dangerous disease” (MDK-1), “[…] a disease that treats one badly […], and a disease that brings you down” (MDK-6). The disease was associated with the personal experience of suffering.

Two interviewees provided explanations for the disease based on tribal spiritual ideas:

I don’t know why I caught tuberculosis, but the spirit walks around the world at night and gets illness. And when it comes back, we get ill and then it becomes tuberculosis […] because the spirit gets illness in the woods (MDK-5).

This description of the ability of the individual’s spirit to leave the body, “wander the world”, and bring back illness points to a close relationship of the Munduruku with the supernatural world of spirits in the woods.

Some narratives expressed denial of interpersonal contagion, especially because it involved close relatives, family, and intra-domiciliary contact: “I don’t know why I had tuberculosis. Dionysus (the husband) had it too. But that is not the reason why I fell ill” (MDK-4).

As two interviewees described, some explanations emphasize the transmissibility of TB, which, however, was attributed to sharing household things:

My deceased husband had it... I got it from him through the pots and spoons (MDK-8); I caught the disease because my brother-in-law had it…, and I used to go to his place for coffee all the time – get the spoon (MDK-3).

The second category considers TB as a personal experience of illness. The interviewees’ personal experience of the disease was analyzed in two dimensions: therapeutic and social.

The therapeutic dimension included the participants’ attitude regarding treatment options, which ranged from adherence to biomedicine to valorization of the traditional indigenous medicine. The former was evident in the visits they paid to village health care centers for assistance, and the latter in the fact that they, preferentially or concomitantly, sought help from local healers and “chest handlers” – lay “specialists” for treating chest diseases, who are considered healers or praying persons to some extent. However, even when the local practices were preferred, the therapeutic itinerary was unlimited to it, as the formal health care system (i.e., the health care centers) was used as a second option:

When I got ill in the chest, I first sought help with the chest handler, and then with the local healer… I didn’t get any better… then, with the nurse (MDK-10). When I got tuberculosis […], I sought help from the local healer […]; then it got really strong, and the solution was to go to the health care unit (MDK-6).

Regarding disease transmission, prevention, and self-care practices during treatment, the narratives highlighted the resort to behaviors that might be traced back to the beginning of the past century. They believed that “avoiding efforts, exposure to the sun, getting cold” (MDK-10); “using separate dishes and cups” (MDK-3); and “not eating certain foods and avoiding walking under the sun or getting wet under the rain” (MDK-9) had an impact on disease control.

The social dimension of TB was detected in the narratives concerning the “pajé brabo” (wild healer), to whom the Munduruku attribute the breakout of sudden or difficult-to-heal illnesses, which might affect a single individual, a group, or the entire community. In such cases, the local healer prescribes the exclusion of the individual identified as the “pajé brabo” from the community. It is believed that this practice eliminates the disease.

Although the *Equipes Multidisciplinares de Saúde Indígena* (EMSI – Indigenous Health Multidisciplinary Teams) have implemented primary care actions, they did not actively search for individuals with respiratory symptoms, nor did they monitor cases treated for TB. Whenever indigenous persons with symptoms suggestive of TB were identified, they were promptly referred to the CASAI at Jacareacanga municipality for investigation of the disease. When TB was confirmed, the patients remained at the CASAI until the end of treatment.

## DISCUSSION

Although some biomedicine aspects were detected in the narratives, most likely resulting from the interaction between health care professionals and religious missionaries, the explanations formulated by indigenous patients in this study to account for TB were based on local traditional notions. As suggested by Helman,^11^ ^,^ ^12^ the interviewees’ perception of TB in this investigation was determined by the context in which it occurs and includes social and cultural features, considering the particularities of this disease.

The explanations attuned to the biomedical logic were identified by the interviewees, who recognized characteristic disease symptoms (such as cough and chest pain), or physical disorders unrelated to TB (such as pain in the legs) in their own bodies. Although leg pain is not a characteristic symptom of TB, the Munduruku believes that the pain can migrate up the body and reach the lungs, where it triggers cough and TB. This is a manner of explaining illness, in which it is depicted as a dynamic process, and different meanings are integrated and combined to (re)create meanings and experiences for illness.^20^

According to Helman,^11^ the concept of disease refers to abnormalities of the structure and function of body organs and systems, which can be identified and described by the reference to certain biological, chemical, or other parameters. Alternatively, illness refers to the subjective response of the patients to being unwell; how they and those around them perceive the origin and significance of this event; how it affects their behavior or relationships with others; and the steps taken to remedy this situation. Therefore, illness is the patients’ perspective on their ill-health, which is a different perspective from that of the disease model proposed by Helman.^11^

Although not prominent, magic-religious explanations could be identified in the narratives about spirits that wander at night and return with illness, and in the description of the intentional action of a specific entity (“pajé brabo”). This denotes a notion rooted in the local culture. Analogous findings have been reported in indigenous populations from the Alto Rio Negro DSEI in Amazonas.^10^ ^,^ ^21^ There, indigenous populations attributed the occurrence of TB to the action of enemies, including poisoning of food, drinks, or personal items; poisons introduced into body orifices; and chants or prayers performed to induce evil.

Similarly, Welch and Coimbra Jr^22^ observed that although the Xavante Indigenous from Pimentel Barbosa, MT, Midwestern Brazil, consider witchcraft to be the main cause of TB, some informants stated that the disease might also be caused by microorganisms. They found that the Xavantes believe in two types of TB, characterized by the speed of its progression: the faster type is caused by spells, and the slower one by microbes. However, according to other informants, TB has only one type and might be caused by either spells or microbes. Therefore, an individual might first fall ill because of a spell and then pass the illness on to others via microorganisms.

Explanations based on traditional indigenous medicine diverge from the notions regarding the transmissibility of TB of the biomedical model, implemented by health care professionals. In traditional indigenous medicine, TB transmission is an illness concept,^11^ associated with indirect contact among people who are socially distant (enemies or adversaries, in particular) in open and public spaces. In a biomedical disease model, the transmission occurs in closed, poorly ventilated, and crowded environments, and is conveyed by direct interpersonal contact, especially within households.

Health care professionals should consider such differences when planning and implementing health actions in indigenous communities. EMSI members should be acquainted with ethnographic characteristics of the targeted populations to provide culturally differentiated care, as recommended by the Brazilian policy for Indigenous health care.^19^

Kleinman and Benson^14^ recommend that all health care professionals, especially those who provide clinical care, should perform a basic ethnographic analysis as part of their activity, summarized in six steps: (i) to identify the ethnic identity of the individual being assessed; (ii) to understand what the individual considers as to be at risk; (iii) to valorize the individual’s illness narrative; (iv) to allow room for the individual to report daily life situations that might disclose conditions of psychosocial stress; (v) to understand the influence of the cultural context on the clinical manifestations; and (vi) to prevent well-meant culturalist approaches from strengthening stigmatization and prejudice that could hinder adherence to treatment.

Although traditional indigenous medicine was predominant among the therapeutic preferences of interviewees, this predominance did not entail the exclusion of biomedicine. The patients’ circulation between the two therapeutic settings is of a complementary nature in the quest for a solution to a health problem, and based on local cultural conceptions and on the use of health care services available at the villages.

The main limitations of this study are related to sampling. The three most populous villages were selected based on feasibility considerations. Moreover, the interviews included the participation of interpreters, which might have hindered the accuracy of the understanding of the narratives, although utmost care was applied when selecting the translators.

The interviewed patients did not separate the magical or religious and the biomedical therapies when affected by active TB. There was simultaneous interaction between two apparently distinct therapeutic systems, both employed for the same purpose, i.e., to get cured. The options were not mutually exclusive; rather, they appeared as parts that interacted without clearly demarcated boundaries.

The EMSI health care professionals should be careful to avoid stigmatizing and prejudicing attitudes when dealing with indigenous patients and local specialists. Moreover, professionals should also attribute appropriate value to the individual perception of the indigenous (as expressed in their illness narratives) and its influence within the local cultural context.

Regarding other ethnic groups, the explanations formulated by the Munduruku for TB are unique, and thus should be valued and considered by policy makers and planners of health care programs. Indigenous-directed health care demands particular attention, especially within the current context of implementing the SUS-affiliated health care networks, due to the cultural plurality of Brazil, the different lifestyles of the indigenous people, and their various degrees of proximity to other groups and the surrounding society.

The scope of health care strategies in indigenous villages remains narrow, as the stationed staff routine does not include an active search for individuals with respiratory symptoms, the performance of sputum cultures, or the investigation of contacts. This precarious situation worsens because the directly observed treatment is not performed in the villages, but the ill individuals are confined to CASAI during the treatment.

It is necessary to review the planning of the health actions implemented in the villages, the training provided to the health care staff, and the availability of services. A revision will pave the way for more effective control of TB in indigenous areas.

## Footnotes

a Ministério da Saúde, Secretaria de Vigilância em Saúde. Especial Tuberculose. *Bol Epidemiol*. 2012;43:1-12. Available from: http://portalsaude.saude.gov.br/images/pdf/2014/julho/23/BE-2012-43-Mar--o---Especial-Tuberculose.pdf

b Ministério da Saúde, Fundação Nacional de Saúde. Relatório de avaliação das morbidades dos distritos sanitários especiais indígenas. Belém (PA): Coordenação Regional do Pará; Fundação Nacional de Saúde; 2010.

c Instituto Brasileiro de Geografia e Estatística. Censo Demográfico e Contagem da População. Rio de Janeiro; 2010 [cited 2013 April 5]. Available from: http://www.sidra.ibge.gov.br/bda/tabela/protabl.asp?c=3175&z=cd&o=7&i=P

d Instituto Socioambiental. Povos Indígenas no Brasil. São Paulo, 2010 [cited 2015 Nov 18]. Available from: http://pib.socioambiental.org/pt/povo/munduruku

e Nogueira LMV. A magnitude da tuberculose e os itinerários terapêuticos dos Munduruku do Pará na Amazônia brasileira [tese]. Rio de Janeiro (RJ): Escola de Enfermagem Anna Nery, Universidade Federal do Rio de Janeiro; 2011. Available from: http://objdig.ufrj.br/51/teses/EEAN_D_LauraMariaVidalNogueira.pdf
